# Micronutrients and nutritional status among children living with HIV with and without severe acute malnutrition: IMPAACT P1092

**DOI:** 10.1186/s40795-023-00774-1

**Published:** 2023-11-02

**Authors:** Mutsa Bwakura-Dangarembizi, Lauren Ziemba, Camlin Tierney, Christina Reding, Frederic Bone, Sarah Bradford, Diane Costello, Renee Browning, John Moye, Tichaona Vhembo, James S. Ngocho, Macpherson Mallewa, Lameck Chinula, Philippa Musoke, Maxensia Owor

**Affiliations:** 1 University of Zimbabwe Clinical Trials Research Centre, Harare, Zimbabwe; 2 Department of Paediatrics and Child Health, University of Zimbabwe College of Health Sciences, Harare, Zimbabwe; 3 Center for Biostatistics in AIDS Research in the Department of Biostatistics, Harvard T.H Chan School of Public Health, Boston, MA USA; 4 Frontier Science Foundation, Amherst, NY USA; 5 FHI 360, Durham, NC USA; 6 IMPAACT Laboratory Center, University of California, Los Angeles, CA USA; 7 National Institute of Allergy and Infectious Diseases, Bethesda, MD USA; 8 NIH, Eunice Kennedy Shriver National Institute of Child Health and Human Development, Bethesda, MD USA; 9 Kilimanjaro Christian Medical University College - Kilimanjaro Christian Medical Center, Moshi, Tanzania; 10 Department of Paediatrics and Child Health, Kamuzu University of Health Sciences, Blantyre, Malawi; 11 University of North Carolina Project Malawi and Department of Obstetrics and Gynecology’s Division of Global Women’s Health, Chapel Hill, NC USA; 12 Makerere University Johns Hopkins University Research Collaboration, Kampala, Uganda

**Keywords:** Malnutrition, Micronutrients, Protein, Albumin, HIV, Nutritional status

## Abstract

**Background:**

Micronutrient deficiencies from malabsorption, gut infections, and altered gut barrier function are common in children living with the human immunodeficiency virus (CLHIV) and may worsen with severe acute malnutrition (SAM). Exploratory data of baseline zinc and selenium levels and changes over 48 weeks in children living with HIV by nutritional status are presented.

**Methods:**

Zinc, selenium, serum protein and albumin levels measured at study entry and over 48 weeks were compared between children aged 6 to < 36 months who were living with HIV and had SAM or mild malnutrition-normal nutrition. Children with SAM were enrolled after 10–18 days of nutritional rehabilitation. Two-sided t-tests were used to compare levels and changes in levels of micronutrients and proteins by nutritional status.

**Results:**

Fifty-two participants, 25 with and 27 without SAM, of median (Q1,Q3) age 19 (13,25) and 18 (12,25) months respectively, were enrolled. Zinc deficiency was present at entry in 2/25 (8%) of those who had SAM. Mean (SD) baseline zinc levels were [52.2(15.3) and 54.7(12.0) µg/dL] for the SAM and non-SAM cohorts respectively while selenium levels were similar [92.9(25.0), 84.3(29.2) µg/L]. Mean changes of zinc and selenium from study entry to week 48 were similar between the children with and without SAM. There was no significant difference between baseline protein levels [75.2(13.2), 77.3(9.4) g/L] and the mean change from study entry to 48 weeks was also similar between the two groups; with a mean difference of 4.6 g/L [95% CI, (-2.4,11.6)].

Children with SAM compared to those without had significantly lower serum albumin levels at study entry with similar levels at 48 weeks.

**Conclusions:**

Children with severe malnutrition who were initiated/switched to zidovudine/lamivudine/boosted lopinavir following 10 to 18 days of nutritional rehabilitation showed normal baseline levels of selenium and zinc, and had comparable selenium levels after 48 weeks. There was a strong positive correlation in entry and week 48 selenium levels within each cohort and for zinc in the non-SAM cohort. These data support the current WHO recommended approach to management of severe malnutrition in CLHIV who are initiated on combination antiretroviral treatment.

**Trial registration:**

Registered with ClinicalTrials.gov Identifier NCT01818258 26/03/2013.

**Supplementary Information:**

The online version contains supplementary material available at 10.1186/s40795-023-00774-1.

## Introduction

Malnutrition underlies nearly 50% of deaths in children below five years of age. Most of these deaths result from the severe form of acute malnutrition (SAM) which presents as both the edematous and non-edematous forms [1]. Around one third of children hospitalized for SAM in sub-Saharan Africa are living with HIV [2]. Most childhood mortality in resource-limited settings results from infectious diseases such as diarrhea, pneumonia, and bacterial sepsis and the vicious cycle of malnutrition and infection is well recognized [3, 4]. HIV infection increases the body’s energy requirements while it reduces food intake and decreases the body’s ability to digest and absorb nutrients leading to malnutrition which in turn accelerates progression of HIV disease [3]. Micronutrient deficiencies associated with HIV [5] may contribute to the pathogenesis of HIV infection through increased oxidative stress and compromised immunity [6]. Without nutritional interventions to increase resistance to infection and disease, improve energy and growth, and achieve a positive response to antiretroviral therapy, CLHIV who have SAM are likely to have nutrient malabsorption and poor outcomes.

Current World Health Organization (WHO) guidelines for treatment of SAM include antibiotics for presumed underlying infections, electrolyte replacement, and nutritional rehabilitation using WHO therapeutic foods for macronutrients and micronutrients replacement. The WHO rehabilitation guidelines do not differentiate by HIV status and starts with stabilization using a low calorie therapeutic enhanced milk-based formula, F-75 (75 kcal and 0.9 g protein per 100 mL) then advances to rehabilitation using another milk-based formula F-100 (100 kcal and 2.9 g protein per 100 mL) or ready-to-use therapeutic food (RUTF) to achieve energy intake of approximately 200 kcal/kg/day once there is clinical improvement and return of appetite [7]. F-75 and F-100 contain 2.0 and 2.3 g zinc per 100 mL respectively, but no added selenium, while RUTF typically contains 11–14 mg zinc and 20–40 µg selenium and 520–550 kcal of energy per 100 g sachet. Mildly malnourished CLHIV may require nutritional supplementation as they may have increased losses of nutrients because of malabsorption and secondary infections. WHO recommends that calories should be increased by 10% for a child living with asymptomatic HIV, 20–30% with chronic illness, and 50–100% with severe malnutrition, until weight is recovered [8].

Micronutrient deficiencies are common in children living in low resource settings and may compound the effects of HIV disease [9]. A 2013 Cochrane review on micronutrient supplementation for CLHIV concluded that vitamin A and zinc supplements are safe and recommended trials on single supplements of vitamin D, zinc, or selenium to contribute to the evidence base of reducing HIV related mortality and morbidity [9].

Selenium is a key component of human selenoproteins that are mostly involved in antioxidant activity and immune function [10]. An association between selenium deficiency, immune dysfunction, progression to AIDS, and death has been shown in cohort studies conducted before antiretroviral therapy (ART) in both children and adults [11, 12]. Zinc is an antioxidant and immune modulator and may have antiviral effects as zinc deficient populations are at higher risk of acquiring viral infections [13]. In adults living with HIV, low levels of serum zinc have been associated with more advanced HIV disease and increased mortality independent of baseline CD4 count [14]. Both zinc and selenium are bound to albumin for their transportation within plasma, and their levels may be low in the setting of hypoalbuminemia, although the total body content may still be normal.

While ART has improved the survival of CLHIV in low resource settings, children often have higher HIV viral loads before initiating ART and take longer to achieve viral suppression compared to adults, independent of ART regimen [15, 16]. In addition, undernutrition is a risk factor for mortality in CLHIV [17] and SAM is associated with worse prognosis and impaired immune recovery in CLHIV on ART [18]. What is not known is the micronutrient status in children with SAM who are living with HIV and whether the micronutrients in the currently recommended WHO nutritional rehabilitation is adequate for these children. The objectives of this analysis were to (1) estimate and compare entry levels of zinc, selenium and albumin in children with HIV and mild malnutrition-normal nutrition to those with HIV and SAM, (2) compare their changes over 48 weeks and (3) assess whether micronutrient levels return to normal over the same period in those with SAM.

## Methods

This is a secondary analysis in the IMPAACT P1092 study, a Phase IV, multicenter, open label, non-randomized study conducted at five sites in four countries (Malawi, Tanzania, Uganda, and Zimbabwe) between October 2015 and September 2017. Details of the main study methods and results from the primary analyses of the pharmacokinetics and safety of zidovudine, lamivudine and lopinavir/ritonavir in children with SAM have been previously published [19]. Briefly, nutritional status of CLHIV aged six to < 36 months at screening was classified using WHO criteria as SAM (Weight-for-height Z-score (WHZ) < -3 or mid-upper arm circumference (MUAC) < 115 mm) or non-SAM (WHZ > -2) [7]. The latter group comprised those with mild malnutrition (WHZ > -2 to ≤ -1) or normal nutrition (WHZ > -1). Children with edematous malnutrition or moderate malnutrition (WHZ -3 to -2) were not included in the study. Children who had SAM were managed according to the WHO guidelines for management of SAM [7] before and during the study screening period and enrolled into the study 10–18 days after starting nutritional rehabilitation. They were given a milk formula (F-75) during the stabilization phase before study entry and subsequently transitioned to another milk formula (F-100). In general, the SAM cohort was recruited from nutritional rehabilitation clinics while the non-SAM cohort was recruited from HIV treatment centers. Participants initiated study-provided ART consisting of liquid formulation of zidovudine, lamivudine, and ritonavir-boosted lopinavir within one day of study entry, with switches from ZDV to abacavir (ABC) allowed in cases of ZDV intolerance or hematologic toxicity.

### Study visits and evaluations

Children were followed through 48 weeks for clinical status and nutritional outcomes at weeks 1, 2, 4, 8, 12, 16, 20, 24, 36, and 48. Blood specimens were collected at study entry and at week 48 for micronutrient analysis (including zinc and selenium) among those participants still on initial study treatment (liquid formulation of ZDV/3TC/LPV/r). Zinc and selenium deficiency were defined as plasma levels below the pediatric reference ranges included in Supplemental Table 1 [20, 21].

**Table 1:** Demographic and study entry characteristics

**Characteristic**	**Severe acute malnutrition**	**Mild malnutrition/normal nutrition**
	***N*** = 25	***N*** = 27
**Sex**		
Male	16 (64%)	13 (48%)
**Age (months)**		
median, (Q1, Q3)	19 (13,25)	18 (12,25)
6- < 18mo	11 (44%)	13 (48%)
≥ 18mo	14 (56%)	14 (52%)
**Cohort subgroup**		
SAM	25 (100%)	0 (0%)
Mild malnutrition	0 (0%)	15 (56%)
Normal nutrition	0 (0%)	12 (44%)
**WHO Clinical Stage**		
Clinical Stage I	8 (32%)	20 (74%)
Clinical Stage II	2 (8%)	4 (15%)
Clinical Stage III	5 (20%)	3 (11%)
Clinical Stage IV	10 (40%)	0 (0%)
**Log**_**10**_** HIV-1 RNA (copies/mL)**		
median (Q1, Q3)	4.8 (4.2,5.6)	5.6 (4.8,6.1)
**CD4% (screening)**		
median (Q1, Q3)	15.0 (9.0,22.6)	23 (17,31)
**Zinc (ug/dL)**		
median (Q1, Q3)	50.4 (41.7,59.8)	53.1 (47.1,62.5)
% zinc deficient	2 (8%)	0 (0%)
**Selenium (ug/L)**		
median (Q1, Q3)	92.7 (77.6,103.9)	83.2 (64.7,105.6)
% selenium deficient	0 (0%)	0 (0%)
**Albumin (g/L)**		
median (Q1, Q3)	35 (31.0,40.0)	41.7 (36.0,47.0)
**Total protein (g/L)**		
median (Q1, Q3)	70.1 (67.1,83.7)	77.0 (73.2,84.0)
**Therapeutic feed**	25 (100.0%)	6 (22.2%)
**Any supplements containing zinc**	8 (32.0%)	4 (14.8%)

Albumin and total protein serum levels were measured at study entry, weeks 8, 16, and 48 and evaluated according to local clinical laboratory reference ranges. Blood for albumin and total protein was collected in serum separator or non-additive tubes and tested using bromocresol and biuret methods, respectively.

### Micronutrient levels

Whole blood specimens for micronutrients were collected in royal blue top EDTA trace element tubes. (BD catalog #368,381; Bectin Dickinson, Franklin Lakes NJ). Specimens were spun at 800xg for 10 min, then plasma was removed and re-spun at 800xg for 10 min. Aliquots were frozen and stored at -70 °C before being shipped to Boston Children's Hospital Laboratory (Boston, MA) for batched testing. Zinc and selenium were measured by a graphite-furnace atomic absorption spectrophotometric method with deuterium background correction and a reduced palladium modifier, using a Perkin-Elmer system [22]. The assay had day-to-day variability < 5.0% for zinc over a wide range of concentrations and for selenium at concentrations of 1.20–2.30 µmol/L.

### Statistical analysis

The SAM and non-SAM cohorts were compared for levels of zinc and selenium at study entry, changes in zinc and selenium levels from entry over 48 weeks, zinc and selenium deficiency at study entry and after 48 weeks, prevalence of hypoalbuminemia (albumin < 35 g/L), and levels of albumin and total protein over 48 weeks.

Baseline values were defined as the closest measurement on or before the day of study entry. Continuous outcome measures were compared between cohorts with a two-sided t-test assuming unequal variance. Differences in the observed prevalence of micronutrient deficiencies and hypoalbuminemia were compared using Fisher’s exact test with mid-*p*-value. Linear mixed effects models compared longitudinal albumin and total protein levels between cohorts. Models included a participant random effect and cohort, follow-up time, and the interaction between cohort and follow-up time as fixed effects. Results were considered statistically significant per analysis plan if the *p*-value was less than 0.05, and no adjustments were made for multiple comparisons. All statistical analyses were performed using SAS 9.4 Institute Inc., Cary, NC,USA.

## Results

**Fig. 1:**
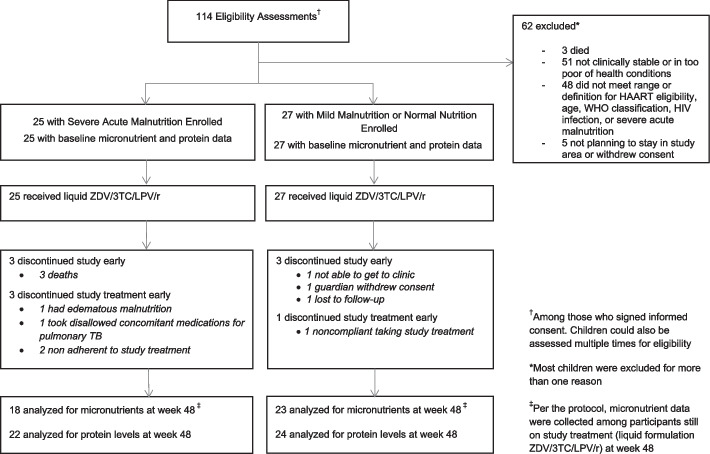
Flow diagram

Fifty-two participants, 25 with SAM and 27 with mild malnutrition or normal nutrition (non-SAM cohort) were enrolled over a 12-month period. A total of 22/25 from the SAM cohort and 24/27 from the non-SAM cohort completed 48 weeks of follow up. Prior to week 48, three (12.0%) SAM participants died, and three (11.1%) non-SAM participants withdrew from the study. Except for one participant who had a low albumin, the three participants who died had normal levels of zinc, selenium, and total protein. All participants living with HIV initiated liquid ZDV/3TC/LPV/r formulations within one day of study entry. Four (16.0%) SAM participants discontinued study treatment early; one developed tuberculosis and another edematous malnutrition while two were non-adherent to study treatment. One (3.7%) non-SAM participant who was non-adherent to ART completed follow-up but discontinued study treatment prior to week 48 and per protocol, week 48 micronutrients were not measured (Fig. 1).

Participants who switched their initial liquid ARV treatment included one SAM participant who switched to ABC/3TC/LPV/r and four participants were switched to a solid formulation of ZDV/3TC/LPV/r for the remainder of follow-up through 48. These five participants had no week 48 selenium or zinc measured but total protein and albumin were measured at week 48.

Table 1 summarizes demographic and study entry characteristics of the 52 enrolled participants. There were more males in the SAM group (64%) and median ages were similar. Fifty-six percent of the non-SAM cohort had mild malnutrition. The median (Q1, Q3) WHZ at screening was -3.4 (-4.0, -3.0) in the SAM cohort and -1.0 (-1.8, -0.1) in the non-SAM cohort. The SAM cohort had a worse WHO clinical disease stage (60% stage III/IV) compared to the non-SAM cohort (11% stage III). Similarly, CD4% at screening was lower in the SAM compared to the non-SAM cohort. Median (Q1, Q3) levels of zinc and selenium were 50.4 (41.7, 59.8) µg/dL and 92.7 (77.6, 103.9) µg/L in the SAM cohort and 53.1 (47.1, 62.5) µg/dL and 83.2 (64.7, 105.6) µg/L in the non-SAM cohort, respectively. At entry, all children in the SAM cohort received some form of therapeutic food compared to 6/27 (22.2%) in the non-SAM cohort, which included zinc. Eight (32%) SAM and 4 (14.8%) non-SAM participants were given zinc through supplements.

After study entry, all children in the SAM cohort received therapeutic food for a median of 20 weeks while 33.3% of the non-SAM cohort took therapeutic food for a median of 6.1 weeks. At week 48, 12/22 (54.5%) of the SAM cohort in follow-up and 4/24 (16.7%) of the non-SAM cohort were taking some form of zinc either as a therapeutic food or supplements (Supplemental Table 2).

**Table 2:** Micronutrient and protein levels at entry and week 48

**Micronutrient**	**Study visit**	**Cohort**				**Differences Between Cohorts**	
		**Severe acute malnutrition**		**Mild malnutrition/normal nutrition**			
		***N***	**Mean (SD)**	***N***	**Mean (SD)**	**Mean Difference (95% CI)**	**(95% CI)** *P*-value^*^
Zinc (µg/dL)	Entry	25	52.2 (15.3)	27	54.7 (12.0)	-2.5 (-10.2,5.2)	0.52
	Week 48						
	Value	18	68.5 (17.2)	23	69.0 (15.6)	-0.6 (-11.1, 10.0)	0.91
	Change from entry	18	15.3 (19.3)	23	15.6 (13.3)	-0.3 (-11.2, 10.5)	0.95
Selenium (µg/L)	Entry	25	92.9 (25.0)	27	84.3 (29.2)	8.6 (-6.5,23.7)	0.26
	Week 48						
	Value	18	93.9 (37.4)	23	83.1 (34.4)	10.8 (-12.3, 33.9)	0.35
	Change from entry	18	-3.2 (22.1)	23	2.0 (25.2)	-5.1 (-20.1,9.8)	0.49
Total protein (g/L)	Entry	25	75.2 (13.2)	27	77.3 (9.4)	-2.0 (-8.5, 4.4)	0.53
	Week 48						
	Value	22	74.8 (6.2)	22	71.9 (4.8)	2.9 (-0.5, 6.2)	0.096
	Change from entry	22	-0.9 (13.4)	22	-5.4 (9.0)	4.6 (-2.4, 11.6)	0.19
Albumin (g/L)	Entry	25	34.7 (6.8)	27	40.9 (7.1)	-6.2 (-10.1, -2.4)	0.002
	Week 48						
	Value	22	43.3 (3.9)	24	43.0 (4.6)	0.4 (-2.2, 2.9)	0.77
	Change from entry	22	8.8 (7.8)	24	2.5 (6.9)	6.3 (1.9, 10.7)	0.006

*SD* Standard deviation, *CI* Confidence interval

^*^Two-sided t-test for the difference in means assuming unequal variance

### Micronutrients

**Fig. 2:**
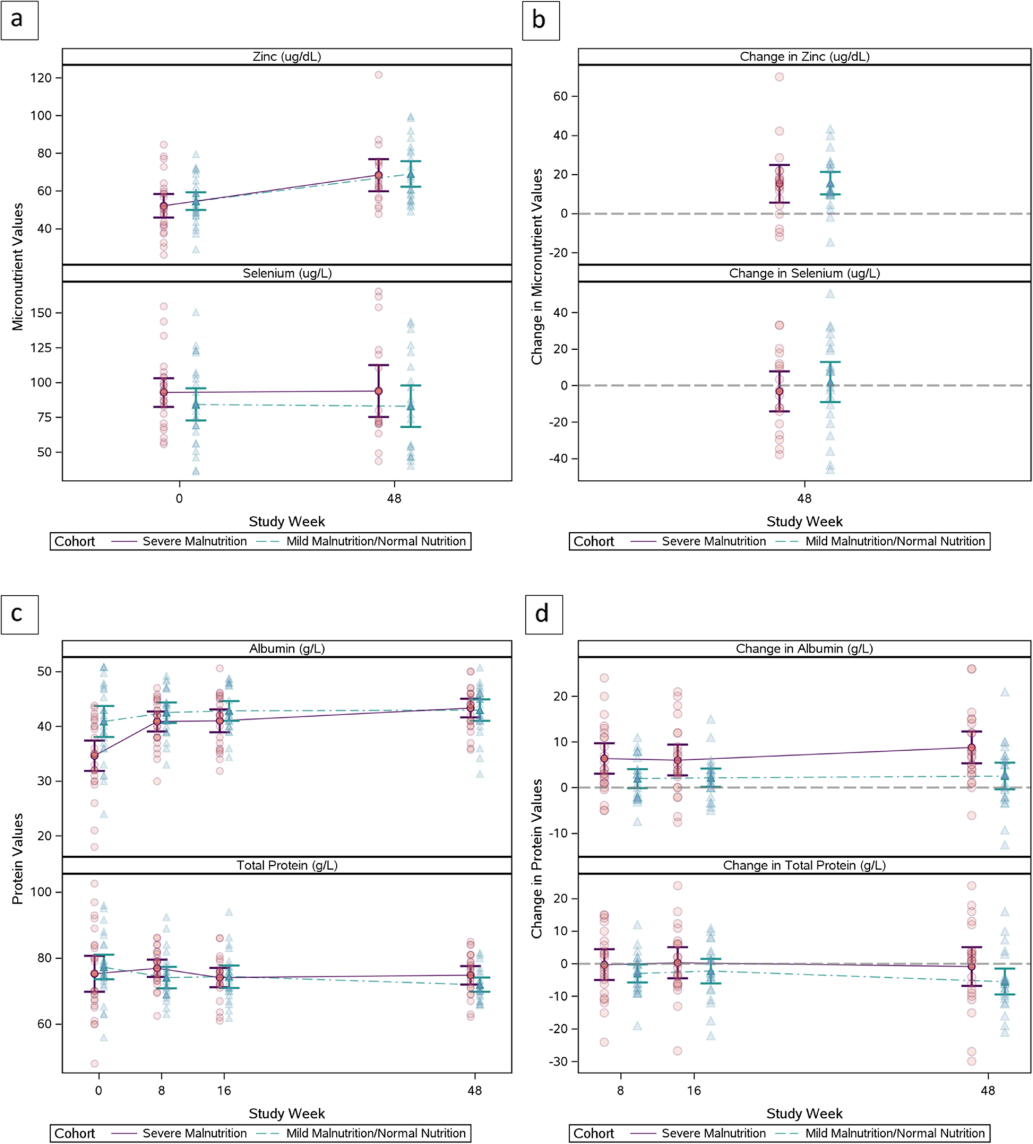
Mean and mean change in zinc, selenium, albumin, and total protein

Table 2 and Fig. 2 show the distribution of zinc, selenium, total protein, and albumin from study entry to week 48. At week 48, 18 (72.0%) participants in the SAM cohort and 23 (85.2%) in the non-SAM cohort were on study treatment and had micronutrient data available.

**Fig. 3:**
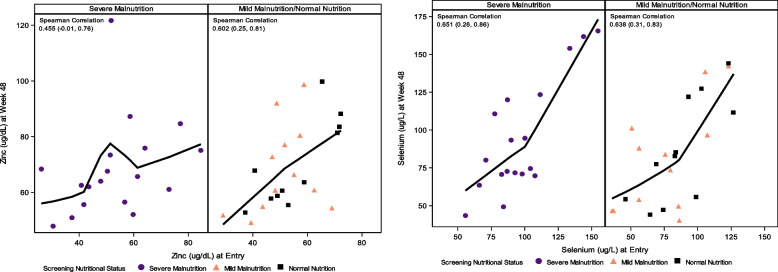
Zinc and Selenium Levels at Entry and Week 48

No participants had selenium deficiency at entry or week 48. At entry, only two participants [(8%) (95% confidence interval (CI) 1.4, 27.5) in the SAM cohort had zinc deficiency and none in the non-SAM cohort (Table 1). Participants with zinc deficiency were one male aged 19 months and one female aged 14 months who took therapeutic feed for 14 days and nine days before entry, respectively. The male participant had a baseline WHZ -3.02, 11.4 cm MUAC, plasma viral load 14,915 copies/mL and screening CD4 percent of 19.6. The female participant had baseline WHZ -4.15, 11 cm MUAC, viral load 182 copies/mL, and screening CD4 23.7%. Both were at WHO Clinical Stage IV at study entry and continued study treatment for 48 weeks. None of the 18 SAM or 23 non-SAM participants with data available had zinc deficiency after 48 weeks of study treatment. At entry, mean zinc levels were similar between the two cohorts (Table 2). At week 48, mean (SD) changes in zinc levels over 48 weeks were also similar: 15.3 (19.3) µg/dL for the SAM cohort and 15.6 (13.3) µg/dL for the non-SAM cohort with a mean difference and 95% CI of -0.3 (-11.2, 10.5) µg/dL. Selenium levels varied but mean levels were similar between cohorts at entry and did not differ after 48 weeks of treatment. The SAM cohort had a mean change in selenium of -3.2 µg/L while the non-SAM cohort had a positive mean change of 2.0 µg/L with a mean difference (95% CI)-5.1 (-20.1, 9.8) µg/L. Overall, there was no evidence of a difference in mean zinc and selenium levels between cohorts at entry or at week 48 nor in the change from entry to week 48 levels. There was a strong positive correlation between entry and week 48 levels for selenium in both cohorts and for zinc in the non-SAM cohort (Fig. 3).

### Total protein and albumin

Mean total protein levels were similar in the SAM and non-SAM cohorts at entry but the SAM cohort had more variability, with mean, (SD) 75.2 (13.2) g/L) and 77.3 (9.4) g/L in non-SAM (Table 2, Fig. 2). The mean change in total protein from entry to week 48 was similar between the cohorts with a mean difference (95% CI) of 4.6 (-2.4, 11.6) g/L; however the SAM cohort had slightly higher levels at Week 48. The SAM cohort had significantly lower albumin levels at entry compared to the non-SAM cohort, mean difference, (95% CI) -6.2 (-10.1, -2.4) g/L but levels were similar after 48 weeks of follow up with a mean difference (95% CI) of 0.4 (-2.2, 2.9) g/L. The mean increase in albumin was significantly higher in the SAM cohort, compared to the non-SAM cohort after 48 weeks (mean difference (95% CI) 6.3 (1.9, 10.7) g/L). Hypoalbuminemia decreased over time in the SAM cohort, from 11 (44%) participants with hypoalbuminemia at entry, two (8.7%) at both week 8 and 16, and none at week 48. (Supplemental Table 3). The non-SAM cohort had five (18.5%) participants at entry with hypoalbuminemia, one (4.3%) at weeks 8 and 16, and two (8.3%) at week 48.

In supplemental repeated measures analyses comparing albumin and total protein levels over all visits (Supplemental Table 4) the SAM cohort had significantly lower albumin levels with an overall mean difference estimate (95% CI) of -2.66 (-4.86, -0.46) g/L (main effects model, *p*-value = 0.02), while no differences were seen in total protein (mean difference estimate (95% CI): 0.37 (-3.25, 3.98) g/L, *p*-value = 0.84). The difference in albumin levels between cohorts decreased over time (estimate of mean decrease (95% CI) in cohort difference: 0.10 (0.04, 0.17) g/L per week, interaction term *p*-value = 0.003).

## Discussion

In this exploratory analysis of micronutrient and protein levels in children aged six to less than 36 months living with HIV, comparing those who had normal or mild malnutrition to severe acute malnutrition, children with SAM had similar micronutrient levels after nutritional rehabilitation and similar albumin levels by 48 weeks compared to those without SAM.

Zinc and selenium deficiency were not common in this cohort of children, unlike an older cohort of ART naive Thai children who all had zinc deficiency at baseline [23]. A similar observation was made in a cohort of 70 ART-naïve Nigerian children, mean age 58 months who had a prevalence of zinc and selenium deficiency of 77.1% and 71.4% respectively compared to 44.3% and 18.6% in HIV negative controls [24]. The duration of stabilization for severely malnourished children (10–18 days) prior to study entry might have improved micronutrient levels of children with SAM thus reducing differences between cohorts at study entry.

The SAM cohort had significantly lower albumin levels at entry, but levels appeared to catch up to the non-SAM cohort by week 48 of follow up. Hypoalbuminemia is a consistent feature of protein-energy malnutrition in children and has been associated with an increased risk of mortality in some studies [25–27].

This study has limitations. The sample size was small, and the analysis was conducted in children after 10–18 days of nutritional rehabilitation, thus missing the opportunity to sample micronutrient and protein levels at initial hospitalization. Micronutrient and protein levels could have improved during the initial period of nutritional rehabilitation prior to study entry. Children with moderate malnutrition were not enrolled in the study by design and results cannot be generalized to this population. Use of therapeutic feeds, other nutritional supplements, or breastmilk containing zinc and selenium in children in the non-SAM cohort may have reduced otherwise expected differences in micronutrient levels between the SAM and non-SAM cohorts. In addition, the study was not able to assess all the dietary intake of the participants.

A strength of this study is that it was conducted in multiple sub-Saharan African countries where acute malnutrition and HIV are highly prevalent, and participants received the same ART regimen. Use of liquid formulations in this study however limits generalizability to solid drug formulations which are in use in most low resource settings in the 6 to the 36-month age range studied.

## Conclusion

In conclusion, this exploratory analysis of CLHIV and malnutrition receiving three-drug combination antiretroviral therapy showed normal levels of selenium and zinc after a period of nutritional rehabilitation and similar albumin levels by 48 weeks of follow up in those with severe malnutrition compared to those with mild-moderate malnutrition. Contrary to the hypothesis, selenium deficiency was not observed and only 8% of SAM participants had zinc deficiency at baseline. Albumin was on average lower in the SAM cohort compared to the non-SAM cohort with normalization to non-SAM levels by 48 weeks. Total protein was similar between cohorts at study entry and through week 48. There was a strong positive correlation between entry and week 48 selenium levels within each cohort and for zinc in the non-SAM cohort. These data support the current WHO recommended approach to management of child malnutrition in CLHIV and severe or mild-moderate malnutrition who receive highly active combination antiretroviral treatment. Attention to nutritional status is a key strategy for both groups to prevent micronutrient deficiency, promote growth, and improve health outcomes.

### Supplementary Information

**Additional file 1:** **Supplemental Table 1.** Micronutrient Reference Ranges. **Supplemental Table 2.** Frequency of Concomitant Mediations by Study Week. **Supplemental Table 3.** Hypoalbuminemia. **Supplemental Table 4.** Longitudinal Analysis of Albumin and Total Protein over 48 Weeks.

## Data Availability

Researchers may submit a request for access to data using the IMPAACT “Data Request” form at: https://www.impaactnetwork.org/studies/submit-research-proposal. Researchers of approved proposals will need to sign an IMPAACT Data Use Agreement before receiving the data.

## Abbreviations

3TC: Lamivudine
ABC: Abacavir
ART: Antiretroviral therapy
CLHIV: Children living with HIV
CI: Confidence Interval
HIV: Human Immunodeficiency Virus
IQR: Interquartile Range
LPV/r: Lopinavir/ritonavir
MUAC: Mid-Upper Arm Circumference
RUTF: Ready to use therapeutic food
SAM: Severe acute malnutrition
SD: Standard deviation
WHO: World Health Organisation
WHZ: Weight-for-height Z-score
ZDV: Zidovudine
