# Supplementary material for: Micronutrients and nutritional status among children living with HIV with and without severe acute malnutrition: IMPAACT P1092
Source: BMC Nutr. 2023 Nov 2;9:121. doi: 10.1186/s40795-023-00774-1 (PMC10621230; doi:10.1186/s40795-023-00774-1)
Supplement: Supplementary file 1 — Additional file 1: Supplemental Table 1. Micronutrient Reference Ranges. Supplemental Table 2. Frequency of Concomitant Mediations by Study Week. Supplemental Table 3. Hypoalbuminemia. Supplemental Table 4. Longitudinal Analysis of Albumin and Total Protein over 48 Weeks. [file 40795_2023_774_MOESM1_ESM.docx]

**Supplementary Material**

**Supplemental Tables for Micronutrients and Nutritional Status among Children living with HIV with and without Severe Acute Malnutrition: IMPAACT P1092**

**Supplemental Table 1. Micronutrient Reference Ranges**

| **Age** | **0-<0.5 years** | **0.5-<1 years** | **1-<2 years** | **2-<4 years** | **4-<6 years** |
| --- | --- | --- | --- | --- | --- |
| **Zinc (ug/dL)^a^** | 26-141 | 29-131 | 31-120 | 29-115 | 48-119 |

| **Age** | **0-< 1 months** | **1-<2 months** | **2-<4 months** | **4-<12 months** | **1-5 years** |
| --- | --- | --- | --- | --- | --- |
| **Selenium (ug/L)^b^** | 14.9-106.3 | 14.9-99.99 | 10.24-92.91 | 13.39-115.75 | 33.86-128.35 |

1. Reference ranges from testing laboratory at Boston Children’s Hospital
2. Ania C Muntau, Monika Streiter, Matthias Kappler, Wulf Röschinger, Irene Schmid, Albert Rehnert, Peter Schramel, Adelbert A Roscher, Age-related Reference Values for Serum Selenium Concentrations in Infants and Children, Clinical Chemistry, Volume 48, Issue 3, 1 March 2002, Pages 555–560.

**Supplemental Table 2. Frequency of Concomitant Medications by Study Week**

|  | | **Cohort** | |
| --- | --- | --- | --- |
|  | | **Severe Malnutrition** | **Mild Malnutrition/Normal Nutrition** |
| **ss** | **Study Week** | **n/N (%)** | **n/N (%)** |
| **Any Zinc Intake (Feed or Supplement)** | Entry | 25/25 (100.0%) | 7/27 (25.9%) |
|  | Week 12 | 22/24 (91.7%) | 5/25 (20.0%) |
|  | Week 24 | 11/22 (50.0%) | 6/24 (25.0%) |
|  | Week 36 | 12/22 (54.5%) | 6/24 (25.0%) |
|  | Week 48 | 12/22 (54.5%) | 4/24 (16.7%) |
|  | **Total** | **25/25 (100.0%)** | **12/27 (44.4%)** |
| **Therapeutic Feed** | Entry | 25/25 (100.0%) | 6/27 (22.2%) |
|  | Week 12 | 20/24 (83.3%) | 4/25 (16.0%) |
|  | Week 24 | 10/22 (45.5%) | 3/24 (12.5%) |
|  | Week 36 | 9/22 (40.9%) | 3/24 (12.5%) |
|  | Week 48 | 8/22 (36.4%) | 3/24 (12.5%) |
|  | **Total** | **25/25 (100.0%)** | **9/27 (33.3%)** |
| **Supplements with Zinc** | Entry | 5/25 (20.0%) | 2/27 (7.4%) |
|  | Week 12 | 3/24 (12.5%) | 2/25 (8.0%) |
|  | Week 24 | 1/22 (4.5%) | 4/24 (16.7%) |
|  | Week 36 | 3/22 (13.6%) | 5/24 (20.8%) |
|  | Week 48 | 4/22 (18.2%) | 2/24 (8.3%) |
|  | **Total** | **15/25 (60.0%)** | **8/27 (29.6%)** |

**Supplemental Table 3. Hypoalbuminemia**

|  | | | **Cohort** | | | |  |
| --- | --- | --- | --- | --- | --- | --- | --- |
|  | | | **Severe Malnutrition** | | **Mild Malnutrition/Normal Nutrition** | |  |
|  | **Study Visit** | **Hypoalbuminemia (<35 g/L)** | **n (%)** | **95% CI** | **n (%)** | **95% CI** | **P-value*** |
| **Albumin (g/L)** | Entry | Yes | 11 (44.0%) | (25.0%, 64.7%) | 5 (18.5%) | (7.0%, 38.7%) | 0.054 |
|  |  | No | 14 (56.0%) |  | 22 (81.5%) |  |  |
|  |  | **Total** | **25 (100.0%)** |  | **27 (100.0%)** |  |  |
|  | Week 8 | Yes | 2 (8.7%) | (1.5%, 29.5%) | 1 (4.3%) | (0.2%, 24.0%) | 0.62 |
|  |  | No | 21 (91.3%) |  | 22 (95.7%) |  |  |
|  |  | **Total** | **23 (100.0%)** |  | **23 (100.0%)** |  |  |
|  | Week 16 | Yes | 2 (8.7%) | (1.5%, 29.5%) | 1 (4.3%) | (0.2%, 24.0%) | 0.62 |
|  |  | No | 21 (91.3%) |  | 22 (95.7%) |  |  |
|  |  | **Total** | **23 (100.0%)** |  | **23 (100.0%)** |  |  |
|  | Week 48 | Yes | 0 (0.0%) | (0.0%, 18.5%) | 2 (8.3%) | (1.5%, 28.5%) | 0.36 |
|  |  | No | 22 (100.0%) |  | 22 (91.7%) |  |  |
|  |  | **Total** | **22 (100.0%)** |  | **24 (100.0%)** |  |  |

**Fisher's mid p-value*

**Supplemental Table 4. Longitudinal Analysis of Albumin and Total Protein over 48 Weeks**

|  | | | | | **Model Estimates** | | | |
| --- | --- | --- | --- | --- | --- | --- | --- | --- |
|  | | | | | **1: Main Effects Model** | | **2: Interaction Model** | |
|  | **Number of Participants** | **Number of Observations** | **Model Type** | **Model Effects** | **Estimate (95% CI)** | **P-Value** | **Estimate (95% CI)** | **P-Value** |
| **Albumin (g/L)** | 52 | 190 | Main Effects Only | Cohort (non-SAM-SAM) | -2.66 (-4.86, -0.46) | 0.02 |  |  |
|  |  |  |  | Study Time (Per Week) | 0.09 (0.05, 0.12) | < 0.001 |  |  |
|  |  |  | Added Interaction Term | Cohort (non-SAM-SAM)*Study Week |  |  | 0.10 (0.04, 0.17) | 0.003 |
| **Total Protein (g/L)** | 52 | 185 | Main Effects Only | Cohort (non-SAM-SAM) | 0.37 (-3.25, 3.98) | 0.84 |  |  |
|  |  |  |  | Study Time (Per Week) | -0.06 (-0.10, -0.01) | 0.02 |  |  |
|  |  |  | Added Interaction Term | Cohort (non-SAM-SAM)*Study Week |  |  | 0.08 (-0.02, 0.17) | 0.11 |

*Acknowledgements*

We gratefully acknowledge the contributions of the site investigators and staff who conducted the IMPAACT P1092 study:

**ZIMBABWE. University of Zimbabwe College of Health Sciences-Clinical Trials Research Centre:** Tapiwa G. Mbengeranwa, MBA^1^; Tsungai P. Mhembere, MPH^1^; Bernadette Malunda, Honours in Biological Sciences^1^. **IMPAACT Laboratory Center:** Carolyn Yanavich, PhD, CCRP^6^; Nancy Tustin, MLT (ASCP) (HEW)^6^. **TANZANIA. Kilimanjaro Christian Medical Centre**: Aisa Shayo MD, MMED^9^; Boniface Njau, MPH^9^; Winfrida Shirima, BSc^9^. **MALAWI**. **University of North Carolina Project-Malawi**: Noel Mumba, Diploma Clinical Medicine^11^; Tionge Kamvaunamwali, NMT, BSPH^11^; Wanangwa Nyirenda, B.Soc.Sc; Wilberforce Mhango, BA, Diploma Pharmacy^11^. **UGANDA**. **MU-JHU Research Collaboration**: Phionah Kibalama, MBChB, MSc PH^12^; Annette Miwanda^12^; Linda Barlow-Mosha, MD, MPH^12^; Irene Lubega, MBChB, MMED^12^. **College of Medicine-Johns Hopkins Research Project**: Dingase Dula, MBBS^13^; Sufia Dadabhai, MHS, PhD^13^; Taha Taha, MD, PhD^13^.
